# Motivation to Avoid Uncertainty, Implicit Person Theories about the Malleability of Human Attributes and Attitudes toward Women as Leaders vs. Followers: A Mediational Analysis

**DOI:** 10.3390/bs14010064

**Published:** 2024-01-17

**Authors:** Federico Contu, Flavia Albarello, Antonio Pierro

**Affiliations:** 1 Department of Social and Developmental Psychology, “La Sapienza” University of Rome, Via dei Marsi, 78, 00185 Rome, Italy; flavia.albarello@uniroma1.it (F.A.); antonio.pierro@uniroma1.it (A.P.); 2 UniSR-Social.Lab, Vita-Salute San Raffaele University, 20132 Milan, Italy

**Keywords:** need for cognitive closure, implicit person theories, attitudes toward women as leaders vs. followers

## Abstract

This research investigated the relation between motivation to avoid uncertainty, as reflected in the need for cognitive closure, implicit theories about the malleability of human attributes, and attitudes toward women as leaders vs. followers. In a cross-sectional study (N = 470) conducted in Italy, we hypothesized and found that the need for cognitive closure directly enhanced the belief that women are compatible with followership roles rather than leadership roles. Furthermore, the results from a mediational analysis revealed that the relation between the need for cognitive closure and the belief that women are compatible with followership roles rather than leadership was mediated by implicit person theories (i.e., the conviction that people features are malleable vs. unchangeable). Notably, we obtained these results while controlling for participants’ gender, educational level, and age. The theoretical and practical implications are discussed.

## 1. Introduction 

The stereotype suggesting an inherent incompatibility between women and leadership roles remains a significant and persistent issue within Western cultures, despite more than five decades of study [1,2]. A substantial body of prior research has uncovered numerous factors contributing to the entrenchment of this phenomenon. Notably, women in leadership positions are frequently assessed as less effective than their male counterparts [3]. Moreover, it is hardly surprising that leadership is predominantly associated with masculine traits in both Western and non-Western societies [4,5]. Consequently, a pronounced bias against women in leadership roles, driven by this stereotype, continues to pervade our society; for a comprehensive review, refer to [6]. Remarkably, recent research conducted by [7] has indicated that in addition to the prevalent bias against women in leadership roles, there exists a preference for women as followers. This implies that the stereotype does not only posit male superiority over women in leadership but also asserts female superiority over men as followers. In essence, the stereotype dictates that the ideal leader should be a man, while the ideal follower should be a woman.

Despite the large body of research conducted on prejudice toward women leaders, the other side of the coin, namely, the prejudice about the compatibility between women and followership roles, has received less attention. Hence, the present research aimed to identify the main psychological catalysts of this kind of attitude. Specifically, embracing a motivational perspective, we investigated the role of (a) the motivation to avoid uncertainty—i.e., the need for cognitive closure [8] and (b) implicit person theories (IPT) [9] as precursors and consolidators of the belief that men are better than women as leaders and women are better than men as followers.

### 1.1. Need for Cognitive Closure, Implicit Person Theory, and Prejudiced Attitudes

The need for cognitive closure (NCC) [8] is defined as a motivation to dislike uncertain knowledge. That is, it is posited that individuals strive for knowledge-certainty and the avoidance of ambiguity when they face new situations. And, particularly, individuals who experience the need for cognitive closure are in search for quick and certain answers to solve the situation that elicited it. Importantly for the present research, when an individual strives for cognitive closure, they will be motivated to search for stable sources of knowledge. Interestingly, the need for cognitive closure can be a dispositional individual difference, or it can be situationally activated through environmental features that induce pressure (e.g., noise or time pressure) see [10] for a review. Notably, those who strive for cognitive closure, also strive for knowledge stability [10]. That is, the high-need-for-closure individuals give certainty to their epistemic processes by embracing and crystalizing certain and simple pills of knowledge. Important to us, the notion that each gender has its stable features and roles (e.g., men are leaders—women are followers) was hypothesized to satisfy the need for cognitive closure [11]. Following this, an empirical examination of this hypothesis confirmed that the NCC (need for cognitive closure) emerged as a pivotal precursor to the reliance on easily accessible schemas [12], which encompass stereotypes [13], resistance to change in well-established environments [14], the perception of racial outgroups as homogenous [15], the expression of attitudes that support the existing system [16], and the display of group-oriented attitudes and behaviors [17]. These behaviors encompass aspects like moral foundations, as seen in [18], as well as the inclination toward strong norms and reduced tolerance for individuals who deviate from established norms, as exemplified in [19,20].

Implicit person theories [9] are grounded in Kelly’s [21] theory of personality and in Heider’s field theory of social perception [22], and specifically refers to people’s naïve assumptions on the malleability of personal attributes. Firstly, the theory posits that people have key implicit beliefs through which they process their social perception of others. It follows that these implicit theories—or beliefs—are separated into two different types of assumptions that individuals may make about the malleability of others’ personal attributes. Specifically, Dweck and colleagues [9], identified the general belief that humans’ attributes are fixed and, thus, that they are non-malleable traits (i.e., entity theory). On the other hand, people may also believe that humans’ main attributes are malleable, and, therefore, that they can be changed, developed, or trained (i.e., incremental theory). Notably, it is interesting to notice that while entity theories represent a stable source of knowledge (more on this point below), incremental theories represent a notion about the possibility that people’s attitudes and beliefs can change and can thus result in a consequent need for certain knowledge.

As we pointed out above, stability is a pivotal feature with respect to knowledge for those individuals that strive for cognitive closure. Hence, it is reasonable to speculate that those who desire cognitive closure and thus dislike instability are more likely to adopt an entity theory in their approach to social perception [23]. In other words, we expect that entity theories of personality that entail people’s attributes’ stability fulfil people’s desire for cognitive closure; meanwhile, incremental theories should frustrate that desire, thereby throwing people into uncertainty. In accordance with Kruglanski’s predictions [23], Levy and colleagues [24] found that a high need for cognitive closure was associated with an endorsement of entity (vs. incremental) implicit person theories. However, these results were not obtained through studies designed to test that hypothesis and they are thus only ‘side results’. Further, there are not, to our knowledge, other studies that have investigated the relationship between these two important constructs. Notably, past research has already indicated a clear association between the need for cognitive closure and the trait-based evaluation of others. For example, Chiu and colleagues [25] found that individuals high in the need for cognitive closure tended to evaluate others in function of naively assumed dispositions. These findings, such as the present hypothesized relation between the need for cognitive closure and implicit person theories, are based on a large body of authoritative research showing that those who naively build their knowledge tend to trace behaviors to a correspondent—stable—disposition; e.g., [26]. As a result, ‘lay’ individuals overestimate cross-situational consistency in humans’ behaviors and have excessive confidence in dispositional and stable traits in predicting trait-related behaviors in specific situations [27,28]. Based on these important findings and arguments, we expected that individuals high in the need for cognitive closure (i.e., who naively build their knowledge) would have tended to rely on entity (vs. incremental) theories of social perception.

Importantly for us, both the need for cognitive closure and implicit person theories have been associated with prejudiced attitudes [10,24]. Specifically in the ‘negative attitudes toward women’ arena, [29] found that individuals with a dispositional need for cognitive closure had higher levels of negative attitude toward women in nontraditional roles. Further, Baldner and Pierro [30] replicated these results with respect to the association between the need for cognitive closure and negative attitudes toward women leaders and found that this relationship is mediated by binding moral foundations—i.e., a concern for the larger group and its norms and standards [31]. Interestingly, a large body of research found other mediators of this process, such as hostile sexism [32], or benevolence toward men [33]. However, research that investigated the mediational role of implicit person theories are lacking.

Furthermore, implicit person theories have been shown to be significantly linked to social stereotyping. Specifically, individuals holding an entity theory, as opposed to those who support an incremental theory, have been found to make more stereotypical trait judgments about various ethnic and occupational groups and tend to evaluate novel groups more extremely. Furthermore, holders of entity theories tend to attribute stereotyped traits to inherent group characteristics rather than situational or environmental factors [24]. In essence, entity theorists tend to engage in a form of lay dispositionism and utilize traits as the fundamental unit for analyzing social perceptions [34]. Consequently, entity theories often disregard mediating factors such as individuals’ intentions to justify their behavior [35] and presuppose that people’s behaviors remain consistent across different time periods and situations [36]. Relevant to the current research, Hoyt and Burnette [37] observed that the correlation between individuals’ attitudes toward women in positions of authority and their subsequent gender-biased evaluations of female leaders is notably more pronounced among those who adhere to entity theories compared to those who espouse incremental theories.

Relevantly, as the need for cognitive closure and entity theories play an important role with respect to the prejudice toward women leaders (i.e., women are incompatible with leadership roles), the same should be true for the reverse. That is, the need for cognitive closure and entity theories should also be associated with the endorsement of the prejudice toward women followers (i.e., women are compatible with followership roles). Indeed, leadership and followership are two sides of the same coin and, as such, it is reasonable to point out that if a category (i.e., women) is considered as incompatible with leadership roles, then it will be considered as compatible with followership roles. Research investigating the compatibility between women and followers is sparse but the few results at our disposal seem to support our idea. For instance, Braun and colleagues [7] showed that individuals that hold implicit theories about how an ideal follower should be associated followership roles with women rather than men. In line with this notion, women, in terms of motivation to gain power, have been always considered as compatible with followership rather than leadership roles [38,39]. Moreover, generally speaking, women do not fit the image of a typical leader and they are thus considered as suitable for followership roles [11,40,41].

### 1.2. The Present Research

To summarize, a large body of research has already investigated the role of cognitive closure and implicit person theories within the gender-based prejudices arena [15,37]. However, to our knowledge, studies that simultaneously investigate the role of cognitive closure and implicit person theories with respect to prejudice toward women leaders do not exist. Further, the other side of the coin (i.e., prejudice toward women followers) has received much less attention. Hence, through a cross-sectional study conducted in Italy, the present research aims to investigate the relation between the need for cognitive closure (i.e., a motivation to avoid uncertainty) and attitudes toward women as leaders and followers. We suggest that those who are characterized by a high need for cognitive closure tend to evaluate women as incompatible with leadership roles and therefore express more negative attitudes toward women as leaders. On the other hand, people who are high in the need for cognitive closure should evaluate women as compatible with followership roles. Furthermore, we hypothesized that the association between the need for cognitive closure and attitudes toward women as leaders vs. followers is mediated by implicit entity theory (i.e., the belief that attributes are fixed). To further test the goodness of our hypotheses, we tested the hypothesized mediational model while controlling for the participants’ age, gender, and educational level.

## 2. Method

### 2.1. Sample Size Determination

To determine the minimum sample size to detect the indirect effects of a simple mediation model, we used the online tool “Monte Carlo Power Analysis for Indirect Effects” by Schoemann, Boulton, and Short [42]. Assuming medium effect sizes (r = 0.30), the confidence level set at 95 percent, the power set at 0.80, 5000 Monte Carlo simulations indicated a minimum sample size of 154 participants to detect the indirect effect of the need for cognitive closure on negative attitudes toward women as leaders (vs. followers) via the implicit person theories.

### 2.2. Participants, Design, and Procedure

To test our hypotheses, we enrolled 470 Italian adults (42.6% males; Mage = 27.40, SDage = 9.15, the age range was from 18 years to 72 years) in a cross-sectional design. With respect to the educational level, 3.0% had a middle school education or lower, 41.3% had a high school education, 53.8% had a university degree, and 1.9% of participants had a Ph.D. Further, 55.7% were students, 40.9% were workers, and 3.4% declared their occupation as “other”. Participants took part in the study, on a voluntary basis, through an online procedure provided by Google Forms. Once they gave their informed consent, participants filled out an online questionnaire aimed at assessing the basic demographic information and the research measures of interest (as described below). Eventually, participants were carefully debriefed and thanked for their participation. The entire questionnaire was administered in Italian; in the following section, we provide the description of the scales we used, and examples of their items in English.

### 2.3. Measures

Need for cognitive closure. Need for cognitive closure was assessed using the Italian version of the Revised Need for Closure Scale (Rev NfCS) [43], a 14-item self-report instrument designed to measure stable individual differences in the need for cognitive closure. Sample items from the scale include statements like, “Any solution to a problem is better than remaining in a state of uncertainty”. Participants indicated their agreement with these statements on a 6-point Likert scale, ranging from 1 (Strongly disagree) to 6 (Strongly agree). The scale demonstrated good reliability with a Cronbach’s alpha of α = 0.74.

Implicit person theory (IPT). Implicit person theory was measured using an 8-item instrument created to assess general implicit person theories about individuals [24,44]. Participants responded to these items on a scale that ranged from 1 to 6, with higher scores indicating agreement with an entity theory (the belief that attributes are fixed), and negative scores representing agreement with an incremental theory (the belief that attributes are malleable). Example items from this scale include, “People can substantially change the type of person they are” and “Everyone is a certain type of person, and there is not much that can be done to really change that”. The IPT scale exhibited excellent reliability with a Cronbach’s alpha of α = 0.93.

Attitudes Toward Women as Leaders and Followers. Attitudes toward women as leaders and followers were evaluated through two items on an 11-point scale [45]. More specifically, to measure attitudes toward women as leaders, we used the scale created by [45]. Meanwhile, to measure attitudes toward women as followers we adapted the scale created by Bhatnagar and Swamy [45]. These scales ranged from −5 (indicating the belief that men are better than women as managers *or* followers) to 0 (indicating the belief that women are as good as men in managerial *or* followership roles) to +5 (indicating the belief that women are better than men as managers *or* followers). To compute a differential measure between attitudes toward women in leadership and followership roles, the scores on the followership item were subtracted from those on the leadership item. Negative scores reflected more negative attitudes toward women in leadership roles and more positive attitudes toward women as followers, suggesting that participants believed women to be superior as followers compared to their roles as leaders.

Control variables. Age, gender (coded as −1 = Male; 1 = Female), and education were included as control variables.

## 3. Results

The descriptive statistics and Pearson’s bivariate correlations are presented in Table 1.

As can be seen, the NCC [r(469) = −0.173; *p* < 0.001] and IPT [r(469) = −0.204; *p* < 0.001] were significantly and negatively correlated with the difference measure of attitudes toward women as managers and followers. Thus, participants with a higher NCC and IPT believed women to be superior to men as followers than leaders. NCC was also positively and significantly associated with IPT [r(469) = 0.206; *p* < 0.001].

**Figure 1:**
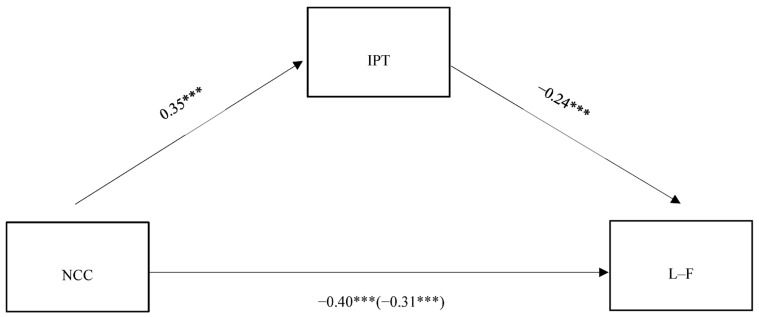
A simple mediation model showing the effects of need for cognitive closure on the prejudice toward women leaders (vs. followers) via implicit person theory. Note: All coefficients are unstandardized. *** *p* < 0.001. Total effect is displayed in parentheses. All effects were obtained controlling by age, gender, and the educational level. Covariates are not included for the sake of clarity. NCC = need for cognitive closure; IPT = implicit person theories (higher score indicate preference for entity theory); L–F = prejudice toward women leaders (vs. followers), (lower score indicate negative attitudes toward women leaders).

To support our hypothesis, we tested the mediating role of IPT in the relation between the NCC and the measure of difference of attitudes toward women managers and followers. Gender, age, and education were included in the model as covariates, which means that their effect was estimated on (a) implicit person theories, and (b) attitudes toward women as leaders and followers. The analysis was performed using SPSS PROCESS macro (Model 4) [46], 95% CIs were employed, and 5000 bootstrapping resamples were run. More specifically, in the model we estimated, the need for cognitive closure was inserted in the estimated model as the independent variable, the implicit person theory was the mediator, and, eventually, the attitude toward women as leaders and followers was the dependent variable. The results obtained from the analysis are summarized in Table 2 (i.e., covariates regression table) and Figure 1.

First, the entire model was significant [R = 0.21, adjusted R-sq = 0.045, MSE = 2.07, F(4, 465) = 5.43, *p* = 0.003]. The analyses revealed that, controlling for gender, education, and age, the total effect of NCC on the difference measure of the attitudes toward women as managers and followers was significant and negative [b = −0.40, SE = 0.1, t = −3.69, *p* < 0.001, (95%CI = −0.61; −0.18)], attesting that the high-need-for-closure participants evaluate women as far superior to men as followers than managers. Moreover, the direct effect of the NCC was also significant when the mediator (i.e., IPT) was included in the model [b = −0.31, SE = 0.11, t = −2.88, *p* = 0.004, (95%CI = −0.52; −0.09)], thus indicating that the effect of the NCC on the attitudes toward women managers and followers was partially mediated by IPT. Further, the NCC was significantly and positively associated with IPT [b = 0.35, SE = 0.08, t = 4.49, *p* < 0.001, (95%CI = 0.20; 0.50)]. IPT, in turn, was negatively and significantly associated with attitudes toward women as managers vs. followers [b = −0.24, SE = 0.06, t = −3.87, *p* < 0.001, (95%CI = −0.37; −0.12)]; meaning that entity theorists (i.e., the belief that attributes are fixed) tend to evaluate women as compatible with followership roles rather than leadership roles. Finally, and more important, the indirect effect of the NCC through IPT was significant (b = −0.08, SE = 0.03, [95%CI = −0.15; −0.03]).

Given the nature of our research, we further tested the moderating impact of gender on the relations between our main variables through running a moderated mediation model (Model 15) [46], where, in addition to the paths estimated in the mediation model described above, we regressed the difference measure of the attitudes toward women managers and followers on the interaction between the mean-centered NCC and gender, and on the interaction between mean-centered IPT and gender. Age and education were included in the model as covariates. Notably, no significant effects of the interactions mentioned above were found. Hence, the analyses revealed that (a) the NCC and (b) IPT had the same effect on the attitudes toward women as leaders and followers for the male and female participants.

Eventually, we also tested two distinct mediational models by considering the participants’ evaluation of the compatibility between a) women (vs. men) and followership roles (model 1a), and (b) women (vs. men) and leadership roles (model 1b) as dependent variables, respectively. Age, gender, and educational level were included as covariates in both the models. Meanwhile, the participants’ evaluation of women (vs. men) as leaders was included as a covariate in model 1a, and the participants’ evaluation of women (vs. men) as followers was included as a covariate in model 1b. These analyses confirmed the results detected in the first model we tested (described above). More specifically, with respect to model 1a, the NCC had a positive and significant a) total [b = 0.36, SE = 0.09, t = 4.02, *p* < 0.001, (95%CI = 0.19; 0.54)] and b) direct effect [b = 0.31, SE = 0.09, t = 3.36, *p* < 0.001, (95%CI = 0.13; 0.49)] on the participants’ evaluation of women (vs. men) as followers. Further, IPT positively and significantly correlated with the participants’ evaluation of women (vs. men) as followers [b = 0.16, SE = 0.05, t = 3.02, *p* = 0.003, (95%CI = 0.06; 0.27)]. Finally, and more important, the indirect effect of the NCC through IPT was significant (b = 0.06, SE = 0.02, [95%CI = 0.02; 0.11]). Along the same lines, with respect to model 1b, the NCC had a negative and significant (a) total [b = −0.22, SE = 0.02, t = −2.34, *p* = 0.019, (95%CI = −0.41; −0.03)], but not b) direct effect [b = −0.16, SE = 0.09, t = −1.64, *p* = 0.102, (95%CI = −0.34; 0.03)] on the participants’ evaluation of women (vs. men) as leaders. Further, IPT negatively and significantly correlated with the participants’ evaluation of women (vs. men) as leaders [b = −0.20, SE = 0.05, t = −3.57, *p* < 0.001, (95%CI = −0.30; −0.09)]. Finally, and more importantly, the indirect effect of the NCC through IPT was significant (b = −0.06, SE = 0.03, [95%CI = −0.12; −0.02]). Given that we measured all the constructs involved in this research through the same methodology, that is, all the measures were obtained through a self-report procedure, we performed the Harmon’s Single Factor test to ensure that our data were not affected by the common method bias. Importantly, the test revealed that only 5.7% (i.e., less than the 50%) of the total variance was explained by the factor into which all the items were loaded. Hence, these findings suggest that our data were not affected by the common method bias.

## 4. Discussion

Drawing from a socio-motivational perspective, this research aimed to clarify the mediational role of the implicit person theory [9] on the effect of the need for cognitive closure [8] on the prejudice about the incompatibility of women with leadership roles and the compatibility with followership roles [47]. More precisely, we hypothesized and found that the need for cognitive closure favors the endorsement of entity theories (i.e., the conviction that individuals’ attributes are fixed and unchangeable), which, in turn, brings people to believe that women’s features are suitable for followership roles but not for leadership roles. Importantly, our results remained significant after controlling for the participants’ gender, educational level, and age. Notably, despite some past investigations that have already established these kinds of associations between the need for closure, implicit person theory, and negative attitudes toward women leaders—e.g., [24,32]—this is the first study that has inquired about the mediational role of implicit person theories within this process. Moreover, this research also enriches the gender-based prejudices arena, since the results confirmed that the same process that brings people to hold that women are incompatible with leadership roles, also brings individuals to support the compatibility between women and followership roles. This information now stands beside a larger body of knowledge which revealed an important number of psychological constructs that lies at the core of the process between the need for closure and negative attitudes toward women leaders—e.g., [31,33,48,49].

### 4.1. Practical Implications

As with the previous research carried out on negative attitudes toward certain groups, our investigation can also offer practical insight in defusing or inhibiting the process that underlies people’s negative attitudes toward women leaders. Firstly, since individuals high in the need for cognitive closure are those who search for simple and stable information, the first practical implication is to train people to endure uncertainty. This could have a double advantage, since, in this way, both the cognitive and the emotional component of prejudice could be reduced, thereby inhibiting the possibility that the prejudice is expressed at a behavioral level [50]. This could be achieved, for instance, through mind exercises like those comprehended in the Buddhist practice of meditation that should solve concern about the negative outcomes of uncertain situations, and instills an attitude of equanimity to whatever comes along. Notably, this kind of training should improve people’s capability of processing useful information (instead of merely simple or stable) while they approach others through social perception. Also, since, as we noted above, the need for cognitive closure is not only a dispositional characteristic but it can also be situationally induced, mind trainings such as the Buddhist ones should be directed to manage situational (i.e., environmental) pressure.

Importantly for the field of negative attitudes toward women leaders, relevant and recent research has demonstrated that the positive relationship between the NCC and negative attitudes toward women as managers was moderated by the quality, but not the quantity, of current or past direct contact experiences with women managers [33]. In other words, fortunately, Allport’s intergroup contact theory [51] seems to work also with respect to prejudice toward women leaders [48]. In this respect, since the present research highlights the mediational role of implicit person theories on the effect of the need for cognitive closure on negative attitudes toward women leaders, future interventions should shed light on the possible positive effect of intergroup contact on changing individuals beliefs from entity theories to incremental ones.

### 4.2. Limits and Future Directions

Our research is surely not without limitations. Firstly, our results rely on correlational data only. As such, we are not able to establish the hypothesized casual consequentiality among the variables we considered. Indeed, despite past research already experimentally demonstrating that the need for cognitive closure enhances people’s tendency to be prejudiced toward women leaders [32], it remains to be ascertained that the need for cognitive closure causes an endorsement of entity theories which, in turn, causes hostile attitudes toward women leaders (i.e., the belief that women are incompatible with leadership roles but compatible with followership roles). Hence, future research should implement longitudinal, or even experimental, studies to assess the casual consequentiality among the variables that we hypothesized.

A notable limitation of our study is the exclusive representation of the participants from a single country, namely Italy. Consequently, the findings of this research cannot be generalized to assert that the process linking individuals with a high need for cognitive closure to prejudiced attitudes through implicit person theories functions in an identical manner across different societies and cultures. It is important to acknowledge that past research has shown a consistent tendency to view women as unsuitable for positions of authority, and there is no indication that this effect is limited to a specific culture; rather, it appears to be a global phenomenon [52,53]. Nevertheless, future research should aim to establish that prejudice toward women in leadership roles is a universally endorsed phenomenon by examining this crucial hypothesis in samples from diverse cultural backgrounds.

## 5. Conclusions

In our research, we conducted an examination of the roles played by the need for cognitive closure, which relates to the motivation to avoid uncertainty, and implicit person theories, which encompass the belief that individuals’ attributes are either fixed (entity theories) or malleable (incremental theories), in shaping negative attitudes toward women leaders. Specifically, we explored the stereotype that perceives women as incompatible with leadership roles but compatible with followership roles. Intriguingly, our hypothesized model retained its significance even after accounting for the participants’ demographic factors, including age, gender, and education. Most notably, gender did not appear to moderate either the effect of the need for closure on implicit person theories or the effect of implicit person theories on negative attitudes toward women leaders. In other words, there was no evidence of a gender-based difference in these associations. Although counterintuitive, these findings align with the established theoretical perspectives regarding the concept of the need for cognitive closure. From a conceptual standpoint, we anticipated that both men and women characterized by a high need for cognitive closure would rely more on the belief that individuals’ attributes are fixed (entity theory). This belief includes the notion of compatibility between men and ideal leadership, as well as women and ideal followership, as these represent sources of stable knowledge.

## Figures and Tables

**Table 1:** Descriptives and bivariate correlation.

	NCC	IPT	Lead	Fol	L–F	EDU	Age	M (*SD*)
NCC	(0.74)							3.40 (0.62)
IPT	0.206 ***	(0.93)						3.15 (1.06)
Lead	−0.011	−0.108 *	—					0.66 (1.45)
Fol	0.169 ***	0.101 *	0.475 ***	—				0.66 (1.41)
L–F	−0.173 ***	−0.204 ***	0.534 ***	−0.490 ***	—			0.00 (1.47)
EDU	−0.042	−0.026	0.001	−0.004	0.005	—		—
Age	0.090	0.007	0.014	0.167 ***	−0.103 **	0.183 ***	—	27.40 (9.14)
Gender	0.044	0.015	0.248 ***	0.121 **	0.085	−0.078	−0.134 **	—

Note: * *p* < 0.05. ** *p* < 0.01. *** *p* < 0.001. Cronbach’s alpha is displayed in parentheses. NCC = need for cognitive closure; IPT = implicit person theories (higher score indicate preference for entity theory); Lead = prejudice toward women (vs. men) leaders; Fol = prejudice toward women (vs. men) followers; L–F = prejudice toward women leaders (vs. followers), (lower score indicate negative attitudes toward women leaders); EDU = educational level; gender coded as −1 = male; 1 = female.

**Table 2:** Regression table showing the effect of each covariate (i.e., gender, age, educational level) on implicit person theories, and the prejudice toward women leaders (vs. followers).

			95% Confidence Intervals				
Dep	Pred	*b*	SE	Lower	Upper	*t*	*p*
IPT	Gender	0.007	0.098	−0.185	0.199	0.071	0.943
IPT	Age	−0.001	0.005	−0.011	0.009	−0.168	0.867
IPT	EDU	−0.029	0.083	−0.197	0.134	−0.345	0.730
L–F	Gender	0.249	0.133	−0.012	0.509	1.868	0.062
L–F	Age	−0.013	0.007	−0.027	0.001	−1.782	0.075
L–F	EDU	0.040	0.112	−0.181	0.261	0.357	0.721

Note. IPT = implicit person theories (higher score indicates preference for entity theory); L–F = prejudice toward women leaders (vs. followers), (lower score indicates negative attitudes toward women leaders); EDU = educational level; gender coded as −1 = male; 1 = female.

## Data Availability

Data are available upon request to the corresponding author.
